# Early Dementia Screening

**DOI:** 10.3390/diagnostics6010006

**Published:** 2016-01-21

**Authors:** Peter K. Panegyres, Renee Berry, Jennifer Burchell

**Affiliations:** Neurodegenerative Disorders Research Pty Ltd, 4 Lawrence Avenue, West Perth 6005, Western Australia, Australia; renee.berry1@my.nd.edu.au (R.B.); jen.burchell@gmail.com (J.B.)

**Keywords:** Alzheimer’s disease, mild cognitive impairment, mild neurocognitive disorder, clinical dementia rating scale, global deterioration scale, amnestic mild cognitive impairment, non-amnestic mild cognitive impairment, cognitive testing, treatments, Solanezumab

## Abstract

As the population of the world increases, there will be larger numbers of people with dementia and an emerging need for prompt diagnosis and treatment. Early dementia screening is the process by which a patient who might be in the prodromal phases of a dementing illness is determined as having, or not having, the hallmarks of a neurodegenerative condition. The concepts of mild cognitive impairment, or mild neurocognitive disorder, are useful in analyzing the patient in the prodromal phase of a dementing disease; however, the transformation to dementia may be as low as 10% per annum. The search for early dementia requires a comprehensive clinical evaluation, cognitive assessment, determination of functional status, corroborative history and imaging (including MRI, FDG-PET and maybe amyloid PET), cerebrospinal fluid (CSF) examination assaying Aβ_1–42_, T-τ and P-τ might also be helpful. Primary care physicians are fundamental in the screening process and are vital in initiating specialist investigation and treatment. Early dementia screening is especially important in an age where there is a search for disease modifying therapies, where there is mounting evidence that treatment, if given early, might influence the natural history—hence the need for cost-effective screening measures for early dementia.

## 1. Introduction

Increased recognition of a prolonged pre-dementia phase in neurocognitive conditions such as Alzheimer’s disease (AD) has led to interest in defining diagnostic definitions and biomarkers to allow for earlier recognition and therefore intervention to prevent or postpone dementia. Although there are currently no specific treatments to block the progression of cognitive decline in AD and other neurocognitive dementias, there are important reasons from a patient’s social and personal perspective that an early diagnosis is important. Early dementia screening by a primary care physician should be completed as soon as possible once a patient or a knowledgeable informant has noticed decline in memory or difficulty in performing day-to-day tasks such as paying bills, shopping or managing medications as this enables opportunities for counseling for future care and a chance to arrange financial and legal matters while decision-making capacity remains.

Conversely, there are a number of potential negative aspects of early dementia screening. There is a small risk of false positives inciting anxiety and/or depression in non-demented people. A positive diagnosis can result in depression, loss of status, loss of employment, loss of driver’s license, acquisition of a stigmatizing label and diminished quality of life [1]. There is also a risk of misdiagnosis of a range of behaviors as dementia, resulting in under-treatment and misdirection of patients to inappropriate services, especially in a multicultural setting where English might not be the primary language [2]. At the extreme, AD diagnosis has also been linked to suicide [3]. Therefore, it should be up to the patient to decide the amount of information they should receive.

## 2. Definition: Early Dementia

Mild cognitive impairment (MCI) is a syndrome defined by a decline in cognition that is greater than the level expected for an individual’s age and education level but that does not interfere notably with activities of daily life (Table 1) [4]. It represents an intermediate state between the cognitive changes of normal aging and the earliest clinical manifestations of dementia, the distinction between which can be quite subtle. In comparison to those with MCI, individuals with early dementia are seen to perform poorly in more than one cognitive domain, leading to a more substantial interference in daily function. Although the definition of mild dementia is indicative of a step towards a progressing disease, there are many similarities in the recognition and diagnosis of MCI and early dementia, so these will be discussed together.

**Table 1:** Diagnosis of mild cognitive impairment (MCI) and mild dementia ^1^ [4].

Criteria	MCI	Mild AD
Evidence of performance	Objective evidence of poorer performance in one or more cognitive domains greater than expected for the patients age and educational background	Objective evidence of poorer performance in more than one cognitive domain such as memory, language, visuospatial or executive function
Interference with daily activities	Limited interference with daily activity; however, complex functional tasks may be completed less efficiently, e.g., preparing meals, shopping alone for clothes and groceries, planning a day’s activity, remembering appointments or paying bills	Significant interference in being able to function effectively at work or during usual activity; however, still able to carry out less complex activity, e.g., ADLs—bathing, dressing and grooming and IADLs—completing chores or attending social functions

^1^ Concern about change in cognition, as compared with previous level based on information from the patient, clinician or corroborative informant. ADLs = Activities of Daily Living; IADLs = Instrumental Activities of Daily Living.

## 3. Diagnostic Criteria

The diagnostic criteria of MCI and early dementia have been greatly derived from the formulation of the diagnostic criteria for AD. Beginning in 1982, two clinical staging systems were published to define the boundaries between normal aging and Alzheimer’s dementia. These are the clinical dementia rating scale (CDR) and the global deterioration scale for aging and dementia (GDS). A suspicion of early dementia was defined by a CDR of 0.5, which allows a person to have mild consistent forgetfulness and doubtful or mild impairment of independent function, where CDR 0 is normal and CDR 1 is mild dementia [5]. The GDS, on the other hand, is another commonly used severity rating scale for dementia that is based on severity of cognitive function with or without subjective complaint [6]. A GDS of 3 can represent MCI or early AD, which is defined as a subtle deficit in cognition and may have some impairment in executive function that affects complex occupational or social activities. Although CDR assesses both amnestic and non-amnestic domains of cognitive impairment, it is heavily weighted towards testing memory and is therefore more appropriate for detecting amnestic mild cognitive impairment (aMCI). Furthermore, it has been found to be suboptimal in recognizing very mild dementia cases [7,8]. Although these are both valuable tools in the rating of severity of dementia, reports in the literature are varied, and their use as diagnostic criteria has affected the validity of studies reporting early dementia.

In 1984, the National Institute of Neurological and Communicative Disorders and Stroke and the Alzheimer’s Disease and Related Disorders Association (NINCDS-ADRA) developed the first diagnostic criteria for Alzheimer’ dementia [9]—defining it as a clinical-pathological entity. The diagnosis of AD could only be “probable” with the presence of cognitive changes, and the confirmation of “definite“ AD that can only be achieved in postmortem examinations. In 2011, the National Institute of Aging/Alzheimer’s Association (NIA-AA) revised these criteria to reflect recent research that indicated that AD affects the brain many years before problems regarding memory, thinking and learning present themselves. These criteria include two new phases of the disease and introduce the utilization of biomarkers in research. Firstly, the introduction of the use of biomarkers would aim to detect pathological changes of AD before the onset of cognitive symptoms—this phase being coined “the pre-clinical phase“ [10]. Secondly, the introduction of a mildly symptomatic but pre-dementia phase, which defines the onset of mild cognitive symptoms in AD with limited interference in day-to-day function, was introduced. Although this does not establish diagnostic criteria that clinicians can currently utilize, it provides guidance for additional research to determine which biomarkers can best confirm Alzheimer’s related changes. Clinical biomarkers such as deposition of Aβ seen on PET imaging were introduced to increase the clinical likelihood of diagnosis of AD on presentation of MCI; however, these are yet to be utilized for routine clinical use [11]. Finally, in 2014, the International Working Group updated their clinical entity of prodromal AD by introducing improved biomarkers for AD and defining a criteria for atypical and non-AD dementia [12], thus recognizing that development of sensitive and specific biomarkers in neurological conditions other than AD are just as important.

Criteria for amnestic mild cognitive impairment include: [13,14].

- Memory complaint, usually corroborated by an informant
- Objective memory impairment for age
- Essentially preserved general cognitive function
- Largely intact functional activities
- Not demented

In 2013, the Diagnostic and Statistical Manual of Mental Disorders (DSM-V) released criteria for a new diagnostic label of dementia as major neurocognitive disorder. This is based upon the knowledge that not all symptoms of dementia indicate amnestic cognitive impairment, and, therefore, this criteria recognizes that memory impairment may not necessarily be the first cognitive domain to be impaired and as such may not indicate the development of AD. The DSM-V now also recognizes a less severe level of cognitive impairment called mild neurocognitive disorder, which closely resembles MCI. It covers all aspects of cognition, which is highlighted by the separate groups amnestic and non-amnestic MCI. The criteria for this diagnosis also reflects that an individual is not affected significantly in daily functioning and enables a higher frequency of dementia diagnosis than the DSM-IV with the aim to reduce stigma associated with dementia and align the guidelines with current clinical practice [13].

## 4. Subtypes

**Figure 1:**
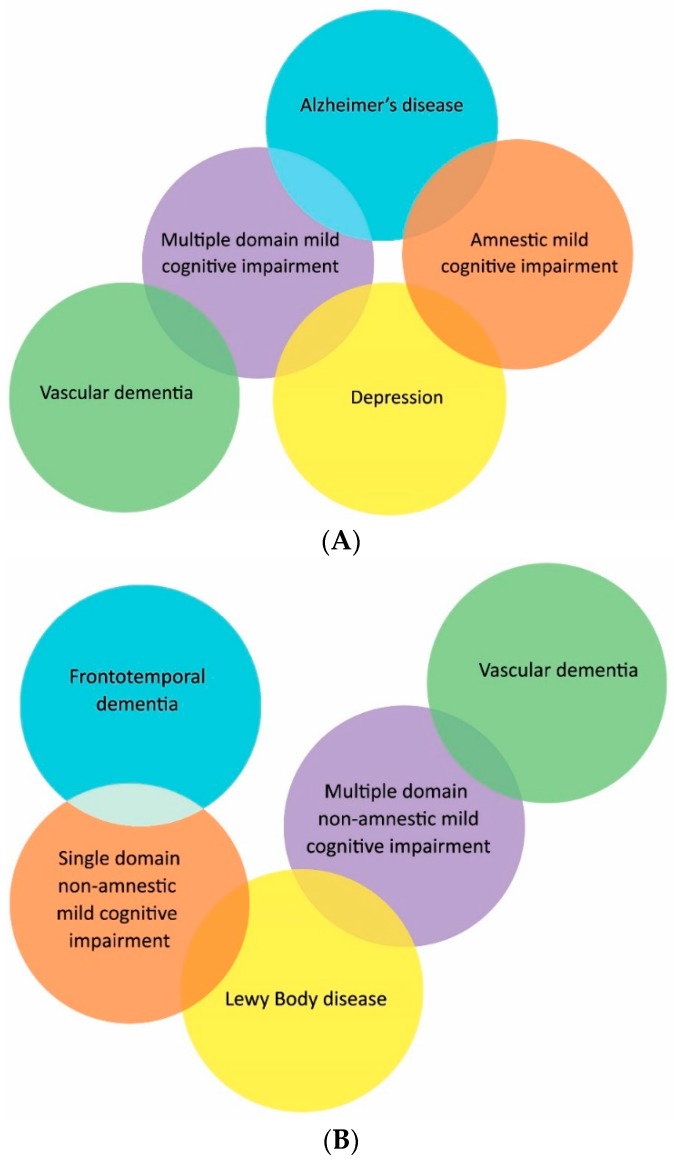
Classification of mild cognitive impairment subtypes with presumed etiology. (**A**) Amnestic mild cognitive impairment; (**B**) non-amnestic mild cognitive impairment.

As previously discussed, MCI was originally referred to as the transition between normal cognitive function and clinically probable AD, which gained widespread use after the introduction of the Mayo criteria for amnestic MCI in 1991 [14]. However, as research on MCI progressed, it had become apparent that MCI is not just a “pre-clinical” state of AD and may not be associated with the development of dementing illness [15]. Based on the International Working Group conference in 2004, new criteria were proposed resulting in four subtypes. These subtypes include amnestic-MCI distinguished by impairment in memory and non-amnestic MCI (na-MCI) where impairment is seen in each of the other central cognitive domains such as language, executive function or visuospatial skills. These can then be further subtyped into whether a single cognitive domain or multiple domains are affected (Figure 1).

As research has progressed, it has become apparent that certain subtypes may be linked to specific dementia etiologies. For example, those with aMCI are more likely to progress to AD; this has been further validated by the presence of biomarkers assumed to be associated with AD, such as changes in MRI, FDG-PET and PiB PET [16]. In the presence of biomarkers, this has greater predictive value; however, these approaches have yet to be validated outside specialized clinic populations. Furthermore, those with na-MCI have a close association with vascular pathology, especially in those with na-MCI multiple domains and a history of vascular disease such as hypertension, ischemic stroke and ischemic heart disease [17]. Mild impairment in multiple cognitive domains indicates a more advanced disease state than a single domain impairment and is more likely to progress to dementia, while those with single domain impairment might revert to normal cognition.

## 5. Epidemiology

Lack of standardized diagnostic criteria for the diagnosis of early dementia has made it difficult to predict which individuals with MCI will progress to dementia. Although clinically based epidemiological studies show a rate of progression of 10%–15% [18], population-based studies have determined a lower risk of dementia progression of approximately 6%–10% per year [19]. Furthermore, population-based studies have determined that up to 44% of patients with MCI at the first visit return to normal a year later [20,21]. It is notable, however, that, in the context of Alzheimer’s dementia, the risk of progressing to AD from MCI in primary care or specialist population setting has an incidence of 1% to 2% per year [20,21]. This finding highlights the importance of obtaining sensitive and specific biomarkers and adequate diagnostic criteria in AD to enable the exploration of the “preclinical phase”. As these diagnostic criteria improve, it would be pertinent to extrapolate these findings to other forms of early dementia.

## 6. Clinical Evaluation

Diagnosis of dementia should be made only after a rigorous clinical assessment, which includes medical history, cognitive and mental state examinations, physical examination and relevant investigations. Furthermore, a review of a patient’s medication profile is imperative to minimize use of medications, which may be adversely affecting cognitive function. Most notably, this should be completed with a knowledgeable informant, as a complete review with an unreliable patient is worthless [22].

More recently, the importance of interviewing a knowledgeable informant has increased [23]. There is evidence that an informant or relative’s assessment of memory correlates more with objective memory scores than a patient’s memory complaints [24,25]. Therefore, it is imperative to obtain an objective memory assessment from an informant or relative. Galvin *et al.* [26] have reported that informant-based assessments provide greater sensitivity than the MMSE in detecting dementia and changes in biomarker profiles of AD, particularly in the early symptomatic stages.

During assessment towards a diagnosis of dementia all aspects of cognitive function should be addressed and, furthermore, an account of the patient’s level of education, prior level of functioning, native language, sensory impairments (especially deafness), psychiatric illness and physical disabilities should be ascertained. Key questions that the medical history appraiser should illuminate are the time of onset, speed of decline and nature of cognitive symptoms. This may give a clue to the potential etiology of the cognitive decline; for example, a rapid cognitive decline is more typical for metabolic disorders, malignancy or prion disease, while the presence of personality changes could allude to the behavioral variant of frontotemporal dementia or traumatic brain injury. Early psychiatric phenomena such as visual hallucinations are observed in Lewy body dementia. Emphasis on the past medical history is important to assess whether the cognitive disorder is transient or due to a delirium or intercurrent illness. The identification of other neurological problems and head injury are important; the presence of cardiovascular risk factors are vital to exclude vascular causes of MCI—vascular dementia being the second most common cause of MCI and dementia in those over 65 years. See Table 2 for a general outline of the clinical differentiation of the common dementias.

### 6.1. Cognitive Testing

A number of screening tests for cognitive decline are available and the mini mental state examination (MMSE) is used widely [27]. To screen for a disease implies the use of a highly sensitive, extremely specific test that can be administered at low cost on large populations of healthy individuals with a very accurate positive and negative predictive value. The MMSE is a test that can be completed in 15 minutes which rates cognitive impairment on a scale from 0 to 30. A review of studies designed to assess the diagnostic accuracy of the MMSE showed that a cut-off score of 27 was indicative of MCI, with a sensitivity of 45%–60% and specificity of 65%–90%, while scores less than 26 indicate worse cognitive function [28]. The Montreal cognitive assessment (MoCA) is a screening tool that was specifically designed for the detection of MCI and takes approximately 10 minutes to administer [29]; it has a sensitivity and specificity of detecting MCI at 80%–100% and 50%–76%, respectively, when using a cut-off of 25/26 [28], which makes it a useful rapid screening test. Furthermore, the realization that the General Practitioner Assessment of Cognition (GPCOG) screen and Mini-Cog testing are as clinically and psychometrically robust as the MMSE [30] implies that these cognitive screening methods might be the way of the future in primary care. These tests benefit in that they are far more streamlined and avoid most of the cultural and language problems of the MMSE.

**Table 2:** Clinical differentiation of the common dementias. Note: AD: Alzheimer’s disease; VD: vascular dementia; LBD: Lewy body dementia; FTD: fronto-temporal dementia; PSP: progressive supranuclear palsy; CBD: corticobasal degeneration.

Disease	Initial Symptoms	Cognitive Impairment	Mental State Examination	Neurological Examination	Imaging Findings
**AD**	Episodic memory loss	Predominance of memory loss with later involvement of all cognitive domains	Initially normal	Initially normal	Entorhinal, cortex and hippocampal atrophy
**VD**	Sudden onset with stepwise deterioration, falls, apathy, focal weakness	Frontal and executive function, generalized slowing, memory may be spared	Apathy, Delusions, Anxiety	Weakness, spasticity, focal neurological deficits	Cortical and/or subcortical infarctions and white matter disease
**LBD**	Visual hallucinations, REM sleep disorder, delirium, Parkinsonism	Drawing and frontal/executive function Spares memory	Delirium, Visual hallucinations, Depression, Delusions	Parkinsonism	Posterior parietal atrophy, larger hippocampi than AD
**FTD**	Apathy, Behavioral and personality change, Poor judgement, Poor speech and language	Frontal/executive, Language, Spares memory and drawing	Apathy, Disinhibition, Hyperorality	May be normal If overlap with PSP/CBD; vertical gaze palsy, axial rigidity, dystonia	Frontal and or temporal atrophy, Spares posterior parietal lobe

General practitioners (GPs) play an essential role in the diagnosis and management of dementia and are the first step in the chain to early diagnosis. In Australia, routine memory tests such as the MMSE, GPCOG or Rowland Universal Dementia Assessment Scale (RUDAS), which is particularly useful for assessing patients from a non-English speaking background, are used by GPs to assess patients with complaints of memory loss. Many of the current brief screening measures, such as the MMSE [27], have modest sensitivity but only fair specificity in detecting dementia [26]. The accuracy of the MMSE depends on a person’s age and educational level; using an arbitrary cut-point (typically < 23) may potentially lead to false positives among people with lower educational levels and false negatives among individuals with higher educational levels [26].

### 6.2. Functional Status

To determine whether a patient meets the criteria for dementia, an investigation of impairment of day-to-day activities is required. The Functional Activities Questionnaire is a brief standardized assessment that allows clinicians to obtain objective evidence from family members or spouses regarding their ability to complete daily activities. Utilizing a cut-off point of 6 points or greater was found to have an accuracy rating of 85% or greater in detecting those with MCI from dementia [31]. The Total Functional Capacity assessment, developed for Huntington’s disease, might also be useful for dementia in general [32].

### 6.3. Review of Medications

A number of medications may confound the diagnosis of cognitive impairment and the risk of polypharmacy, especially in the elderly, can further make assessment difficult. Therefore, a full review of all prescribed and over-the-counter medications should be completed. Some of the most important drug classes which may be a factor in cognitive impairment include opioids, anticholinergics, tricyclic antidepressants, benzodiazepines and non-benzodiazepine hypnotics, muscle relaxants, antihistamines and antiepileptics. Furthermore, a thorough history of alcohol intake, cigarette smoking and illicit drug usage is essential.

### 6.4. Neurological Evaluation

A complete neurologic evaluation is required, including an assessment of vision and hearing. A speech impediment might indicate the presence of Parkinsonism, while gait disturbance might allude to Parkinsonism, FTD, normal pressure hydrocephalus or stroke. Always consider the presence of a space-occupying lesion, such as tumor or subdural hematoma, in the case of indolent morning headaches, or patients with lateralizing signs on cranial nerve examination as a cause of cognitive decline. The peripheral nervous system should be examined in order to detect neuropathy due to toxins or nutritional problems such as diabetes or vitamin deficiencies. A past history of head injury or neurological disorders must be determined, as traumatic brain injury and epilepsy [33] are risk factors for the development of early cognitive decline. Finally, it is important to address changes in sleeping patterns as this might lead to cognitive symptoms—sleep apnea and restless legs syndrome must be considered; sleep disorders can also result in depression, which can further compromise cognition. Treatment of these disorders may improve attention, which will allow for an assessment of whether cognitive impairment due to a degenerative disorder has developed.

### 6.5. Psychiatric Evaluation

The completion of a focused mental state examination for psychiatric symptoms should be completed in all patients who have MCI. An evaluation of mood and thought disturbance may indicate depression as a cause for decline in cognition. Utilizing the Geriatric Depression Scale can aid in recognition of depressive symptoms in elderly patients, with a score greater than 6 indicative of depression [34]. This scale needs to take into account the patient’s cultural and language background. Visual hallucinations might suggest dementia with Lewy bodies, and behavioral or personality change may indicate the presence of frontotemporal dementia or psychiatric illness. Paranoid delusions may be common in those with significant forgetfulness and impaired judgement; therefore, it is important to elucidate whether these manifestations are due to cognitive problems or psychiatric illness.

### 6.6. Social History

In order to classify a change in a person’s level of cognitive function, it is imperative to determine a person’s social history. Certainly, one’s level of education plays an important factor as well as occupation, hobbies, home life and supports.

### 6.7. Additional Testing

A full routine blood workup is important to exclude underlying conditions such as infection, uremia or liver disease. Furthermore, thyroid function, B12, folate and fasting glucose are important to exclude metabolic and nutritional deficiencies. Toxicity to medications (such as antiepileptics or digoxin) must also be considered. If risk factors are present in a patient’s medical history, testing for sexually transmitted infections is important, especially syphilis and HIV.

Structural imaging, preferably MRI, is necessary to exclude any potentially reversible causes of dementia such as stroke, intra-axial and extra-axial tumors, subdural hematomas and hydrocephalus [35]. According to UK, European and American guidelines structural imaging should occur at least once during the initial investigation of dementia, utilizing non-contrast CT or MRI, with MRI being the preferred technique [36]. It is well known that MRI has a higher resolution than CT and enables detection of more subtle anatomical and vascular changes [37]; however, a review of 38 studies comparing MRI to CT for investigation of vascular dementia determined that MRI was no more superior in detecting vascular dementia [38]. The overall sensitivity and specificity of MRI in the detection of space occupying lesions is 88.9% and 91.9%, respectively, where CT is 80.1% and 85.4% respectively [39]. A clinician’s decision as to which imaging modality is chosen may also be limited to contraindications to MRI such as pacemaker insertion and relative contraindications such as an inability to lay still or claustrophobia. Furthermore, availability and financial costs may limit use of MRI, especially in rural centers. With respect to AD, the typical finding on MRI is global brain atrophy with early disproportionate symmetrical involvement of medial temporal lobe structures such as the hippocampi [40]. Medial temporal lobe atrophy has been found to predict which individuals will develop clinical AD from MCI with sensitivity and specificity of 73% and 81%, respectively, [40] and, furthermore, differentiates AD from normal aging with a sensitivity and specificity of around 80%–85%.

To enable differentiation between subtypes of neurodegenerative dementias Positron emission tomography (PET) using 18-F-flurodeoxyglucose (FDG) and SPECT using 99mTc-hexamethylpropyleneamine oxime (99mTc-HMPAO) are available and characterize subtypes by assessing neuronal function. PET has been shown to be superior to SPECT, although this evidence is limited [41] and has the benefit of providing greater functional resolution of deeper structures and allows for use of radiotracers specific to dementia subtypes. A meta-analysis comparing FDG-PET to other methods of AD diagnosis such as clinical guidelines, MRI, SPECT and other biomarkers has determined that PET imaging is superior [37] with a 91% and 86% specificity and sensitivity, respectively [42]. PET accurately distinguishes normal patients from MCI [43], and, furthermore, the specific amyloid radiotracers 11C-PiB have been found to accurately predict short-term conversion of MCI to AD with a sensitivity and specificity of 93.5% and 56.2%, respectively [44]. The use of amyloid PET imaging is limited in the community due to the costs of the ligand tracer and imaging equipment and not recommended in cognitively normal elderly subjects due to the presence of false positives from physiological brain amyloid deposition [10]. Furthermore, the routine use of SPECT imaging is not recommended, as a normal SPECT does not exclude AD [42] and has little value over a standard comprehensive clinical assessment when trying to differentiate MCI from dementia.

Amyloid-β_1–42_ (Aβ_1–42_) is the most abundant species found in plaques and was therefore a logical target for the development of screening assays [45]. It is a product of normal cell metabolism and is secreted into CSF. Patients with AD have an approximate 50% reduction in CSF Aβ_1–42_ compared to healthy controls making it the most useful single marker for predicting patients with AD [46]. To date, the most sensitive and specific CSF biomarker combination for the detection of dementia is the triumvirate of a low Amyloid β1–42 (Aβ_1–42_), a high Total-τ (T-τ) and Phosphorylated-τ (P-τ) [46]. The combination of the three biomarkers increases the sensitivity and specificity of diagnosis over use of any of the three biomarkers alone [46], and might be useful in detecting early dementia.

### 6.8. Final Assessment

Utilizing the above guidelines, the physician needs to determine whether the patient has (i) no cognitive impairment, (ii) cognitive impairment without dementia, or (iii) dementia. Those without impairment reassurance should be provided with follow up for re-evaluation in approximately 6 months or earlier if there is a significant change function. Currently, serial cognitive assessments are the most useful markers for changes in cognition as they provide the clinician with a baseline and trajectory of cognitive function over time. A recent review on screening for cognitive impairment has determined that brief cognitive assessments have a low sensitivity for identifying MCI in primary care despite being useful for detecting dementia [47]. With those found to be cognitively impaired but without dementia, bi-annual follow-up appointments should be initiated with the advice that currently it is unknown whether those with MCI will progress to dementia; however, their risk is greater than the general population. Furthermore, as the use of biomarkers such as MRI, FDG-PET and CSF analysis are currently only utilized in the specialist research setting, standardized cut-off points for their interpretation are yet to be determined. Thus, the exact timeframe when to repeat testing in individuals with positive biomarkers is yet to be specified. Experts currently do not recommend that neuroimaging or CSF assays should be conducted in individuals who are asymptomatic or clinically normal; however, ongoing population-based studies may reveal their potential use as a screening method in the future. With those with evidence of cognitive impairment and positive biomarkers, specialist input should be initiated in the unlikely case that it has not already.

## 7. Possible Treatments

### 7.1. Anticholinesterase Inhibitors

Acetylcholinesterase inhibitors are used for mild to moderate AD (donepezil, rivastigmine, galantamine) and an NMDA glutamate receptor inhibitor (memantine) for moderately severe AD. Acetylcholinesterase inhibitors might not be beneficial for mild cognitive impairment [47]. There is evidence that cholinesterase inhibitors are not as successful if treatment is delayed by 6 months; however, the time from the presentation of symptoms to a GP to an accurate diagnosis can be up to 12 months or longer [48]. These medications might have some effect on delaying the progression of disease but do not target the underlying pathology and have side effects.

### 7.2. Monoclonal Antibody Treatment

#### Solanezumab

Solanezumab is a humanized monoclonal antibody directed against the mid-region of Aβ (Aβ_13–28_) and various N-terminal truncated Aβ species such as Aβ_3–42_ [49]. It was designed to bind Aβ monomers in the brain and allow them to be degraded in plasma and CSF. In Phase I and II of the clinical trials [50,51], Solanezumab treatment was associated with increased unbound CSF Aβ_1–42_ and decreased unbound Aβ_1–40_, indicating that this treatment was potentially destabilizing Aβ equilibria to mobilize Aβ_1–42_ from amyloid plaques [51]. Additionally, there was no evidence of meningoencephalitis or other side effects up to one year of follow-up [50,51]. These encouraging results led to two large randomized, double-blind, controlled Phase III trials of Solanezumab called Expedition 1 and Expedition 2, each enrolling over 1000 mild to moderate AD patients [52]. However, these two trials did not meet primary and functional end points. Reanalysis of this data showed that there might be a benefit in patients with mild early dementia (this is the subject of the Expedition 3 project currently in progress).

A major limitation of the recent clinical trials of Solanezumab is timing. It is possible that these trials may have failed to reach their clinical endpoints because the disease was too far advanced for the treatment to have a significant effect. It is now believed that AD might start 15–20 years before symptoms begin. Additionally, there is speculation that only around 1% of the antibodies crossed the blood-brain barrier, so only a small proportion had access to Aβ in the brain.

Clinical trials are currently in progress to assess whether higher doses of Aβ antibodies can prevent cognitive decline in pre-symptomatic patients with less heterogeneous disease. Both the Alzheimer’s Prevention Initiative and the Dominantly Inherited Alzheimer Network’ (DIAN) will assess new Aβ antibodies in pre-symptomatic patients with autosomal-dominant gene mutations which result irrevocably in AD. The Anti-Amyloid Treatment in Asymptomatic Alzheimer Disease will test Solanezumab in mildly symptomatic Alzheimer’s patients who have had PET scans revealing plaque deposits but show no signs of cognitive decline [53]. Other monoclonal antibodies targeting Aβ are also in development (e.g., Aducanumab).

### 7.3. Tau

Epothilone D is a microtubule-stabilizing agent and has been used in a number of prevention and intervention trials to replace tau sequestered into tangles. A mouse model study reported that Epothilone D reduced tau pathology whilst improving axonal transport and memory impairments. A recent mouse model utilizing PS19 tau transgenic mice, who develop tau pathology by six months of age, has demonstrated that this compound can cross the blood-brain barrier, resulting in improved CNS microtubule density and axonal integrity without notable side-effects [54]. Unfortunately, this treatment did not dramatically reduce tau pathology. Leuco-methylthionium bis (hydromethanesulfonate) (LMTM), a methylene blue analogue and currently under investigation for AD and FTD—TauRx 007 and 015 trials—prevents tau aggregation but has not been used in MCI or early dementia. Treatments targeting tau might be useful in the future for early dementia.

### 7.4. Non-Pharmacological Treatments

#### 7.4.1. Exercise

It is well known that AD adversely affects patients’ cognitive, emotional and behavioral functioning [55]. Additionally, AD can adversely affect physical abilities leading to a loss of independent mobility [56,57]. Exercise can increase cardiovascular fitness, reduce the risk of falls and reduce depression [58], and might help improve cognition in early dementia.

#### 7.4.2. Cognitive Training

The brain has the potential to reorganize itself and experience functional improvement after damage [59]. A number of studies have reported that cognitive training can reduce memory impairment. Cognitive training includes cognitive stimulation, memory rehabilitation, reality orientation, and neuropsychological rehabilitation [60]. Cognitive impairment is related to multiple factors including intelligence, education, occupation, methods for processing tasks, coping skills for stressful experiences and choice of cognitive leisure pursuits. A randomized controlled trial of 86 mild AD patients reported that cognitive training in conjunction with donepezil increased patients’ MMSE score over cognitive training or treatment alone at the one-year follow-up [61]. Using functional MRI, patients with early-stage AD showed increased activation of a number of cortical areas whilst engaged in cognitive tasks, suggesting a functional compensation for neuronal loss [62]. Together, these studies suggest that cognitive training might delay or even partially reverse cognitive impairment seen in early AD.

## 8. Conclusions

Even though there are difficulties with early dementia screening, we believe that it is important to recognize the early phases of dementia so that the diagnosis can be established and the treatments introduced. We are in an era of treatments, particularly monoclonal antibodies, for dementia, and there is emerging evidence that the earlier this treatment is administered, the more potential there is for a better outcome. Screening should be considered by primary care physicians, with quick referral to specialists to confirm the possibility of early dementia, the instigation of investigations and the initiation of treatment. This should be done in a caring and supportive manner with regular follow-up and assessment.
